# The Use of MR-Guided Radiation Therapy for Head and Neck Cancer and Recommended Reporting Guidance

**DOI:** 10.1016/j.semradonc.2023.10.003

**Published:** 2024-01

**Authors:** Brigid A. McDonald, Riccardo Dal Bello, Clifton D. Fuller, Panagiotis Balermpas

**Affiliations:** * Department of Radiation Oncology, The University of Texas MD Anderson Cancer Center, Houston, TX; † Department of Radiation Oncology, University Hospital Zurich and University of Zurich, Zurich, Switzerland

## Abstract

Although magnetic resonance imaging (MRI) has become standard diagnostic workup for head and neck malignancies and is currently recommended by most radiological societies for pharyngeal and oral carcinomas, its utilization in radiotherapy has been heterogeneous during the last decades. However, few would argue that implementing MRI for annotation of target volumes and organs at risk provides several advantages, so that implementation of the modality for this purpose is widely accepted. Today, the term MR-guidance has received a much broader meaning, including MRI for adaptive treatments, MR-gating and tracking during radiotherapy application, MR-features as biomarkers and finally MR-only workflows. First studies on treatment of head and neck cancer on commercially available dedicated hybrid-platforms (MR-linacs), with distinct common features but also differences amongst them, have also been recently reported, as well as “biological adaptation” based on evaluation of early treatment response via functional MRI-sequences such as diffusion weighted ones. Yet, all of these approaches towards head and neck treatment remain at their infancy, especially when compared to other radiotherapy indications. Moreover, the lack of standardization for reporting MR-guided radiotherapy is a major obstacle both to further progress in the field and to conduct and compare clinical trials. Goals of this article is to present and explain all different aspects of MR-guidance for radiotherapy of head and neck cancer, summarize evidence, as well as possible advantages and challenges of the method and finally provide a comprehensive reporting guidance for use in clinical routine and trials.

## Background

Radiotherapy (RT) is a standard treatment modality for many head and neck cancers (HNC) but poses significant challenges for many patients due to radiation-induced toxicity to critical organs and structures. In recent years, magnetic resonance imaging (MRI) has become increasingly utilized in radiation therapy applications such as target delineation, treatment planning, and on-board imaging and has shown particular advantage for HNC as a use case due to its excellent soft-tissue contrast. This paper will review the historical context, current technology, and published literature on MR-guided RT (MRgRT) for HNC and will explore its potential to improve outcomes for HNC patients.

The first efforts to integrate MRI into RT workflows were undertaken shortly after its clinical implementation in the 1980s, mostly for target definition of brain tumors.^1^ The inherent features of MRI, mainly the improved soft-tissue contrast, led to its rapid adoption for HNC. As one of the first clinical examples, Curran et al.^2^ demonstrated in 1986 that boost volumes differed significantly from those generated only with computed tomography (CT) in patients with nasopharyngeal carcinoma. With the emergence of highly conformal and high-precision RT techniques such as intensity-modulated radiotherapy (IMRT) in the 1990s, the need for more precise target definition, especially in areas with close proximity to critical organs at risk like the head and neck region, became more urgent than ever. However, due to the limited availability of MRI and technological barriers such as computing capacities and still-unsolved problems like handling image distortions and lack of electron density information, the use of MRI for HNC RT was restricted for a long time to providing information for target delineation supplemental to CT.^3^ In recent years, innovations in MRI hardware and software, the advent of artificial intelligence (AI), and the increasing availability and reduced costs of MRI have led to a broader utilization of this technology, even in middle- and lower-income countries, but also to surmounting technical and physical challenges.^4^

Nowadays, MR-guided RT (MRgRT) is a term with a much broader meaning, encompassing various procedures and possibilities, such as utilizing MRI at the stage of simulation with or without an additional CT scan, registering different sequences together with CT and/or PET imaging, generating synthetic CTs, using MRI for plan adaptation, performing on-line adaptive treatments with or without 4D-gating and tracking, and using specific imaging, radiomic analysis and delta-radiomics to predict tumor response and normal-tissue toxicity. In particular, the development of novel hybrid platforms combining linear accelerators (linacs) with on-board MRI scanners, so-called “MR-linacs,” paved the way for various new opportunities including daily on-table treatment adaptation, monitoring inter- and intra-fractional motion, complete omission of CT in RT workflows, and longitudinal evaluation of quantitative imaging biomarkers during treatment. Although the added value of MRI for target delineation in HNC has been acknowledged and recommended in most national and international guidelines,^5–7^ no consensus currently exists about any of the other aforementioned aspects of MRgRT, especially regarding to the reporting of adaptive treatments. Furthermore, MR-linac technology in general still remains in development and, with its clinical implementation for HNC confined to a few academic centers, there is currently a lack of established clinical evidence to support its widespread use for this indication.

The goal of this review is to address current developments and challenges, with a focus on adaptive approaches, and to provide a guide for reporting MRgRT treatments in order to facilitate both a broader clinical implementation and the generation of robust scientific evidence in the near future.

## MR for Treatment Planning

### MR-Based Segmentation and Auto-Contouring

MR imaging provides improved soft-tissue visualization compared to CT-only approaches, allowing for more precise delineation of HNC gross tumor volumes (GTVs).^8–12^ Rasch et al.^9^ and Cardenas et al.^13^ concluded that implementation of co-registered MR-sequences could reduce inter-observer variability in target delineation in patients with oropharyngeal carcinoma and other head and neck malignancies compared to CT-based contouring alone and that MR-derived GTVs were generally smaller. Still, large differences continue to exist between experts, at least when only simple T1 and T2 sequences are used. In contrast, Ligtenberg et al.^14^ did not observe target volume reductions using MRI for contouring larynx and hypopharynx tumors when compared to CT and PET, although the volumes were still smaller when compared with a geometrical expansion of 10 mm. The same research group compared their MRI-derived GTV segmentations based on laryngectomy surgical specimens and intriguingly found that the MR-based volumes delineated in clinical routine were twice as large as the corresponding pathologically defined tumor volumes.^15^ Therefore, they stressed the urgent need for validated delineation guidelines to avoid such overestimations, although to this day no such international consensus exists. In addition to the delineation of primary tumors, MRI can also help with lymph node segmentations. A recent planning study achieved improved organs at risk sparing through an innovative concept of MR-guided elective nodal irradiation, targeting only individual lymph nodes.^16^ Finally, segmentation recommendations for several organs-at-risk based on MRI have been developed and could guide delineation of those structures, taking into account the improved soft tissue boundary visualization of MRI.^17^ Incorporation of MRI could also be beneficial for organs like the pharyngeal constrictor that are not clearly visible on CT,^18^ and different groups have been developing such contouring guidelines.^19^

With the broader availability of MR-imaging for RT planning and its continuous integration in the treatment planning process, first efforts have been made to replace CT-atlas-based auto-contouring of OARs and nodal levels. After building an atlas-library from T1-images of 12 patients with OARs contoured by a human expert, Kieselmann et al.^20^ investigated the accuracy of automatic MR-based planning, discovering exceptional geometric accuracy, although there were significant dosimetric differences. However, the treatment plans did achieve the clinical goals. As expected, MR-atlas-based auto-segmentation leads to superior results compared to CT, especially for organs like orbits, parotid glands, brain-stem, and the nodal level II.^21^ These benefits of MR applications in OAR delineation soon led to the implementation of novel machine learning methods for MR-based auto-segmentation, taking the next step compared to expert-derived guidelines and atlas-based auto-contouring. First works on utilizing convolutional neural networks for this purpose in HNC have already been developed,^22–26^ even using low-field data (0.35 T),^27^ demonstrating the feasibility of AI-derived, fully automated segmentation. These algorithms result in high accuracy and reproducibility, and many already outperform established models. Moreover, the extraordinary speed of the procedure (< 1 minute) can enable easy integration into a daily on-line adaptive workflow.^23^ Such approaches will facilitate on-line segmentation and can substantially speed up on-line adaptive RT (ART) for HNC. While deep learning models trained on MR images have shown promise for auto-segmentation, large training sets of segmented MRI data are not yet widely available. A number of alternative approaches have been proposed that use CT images to augment MRI-based auto-segmentation models. For example, Dai et al. and Kieselmann et al. have developed models that generate a synthetic MRI from CT or cone beam CT (CBCT) imaging to artificially improve soft tissue contrast, which is then used for OAR auto-segmentation to aid treatment planning and/or ART of HNC on conventional linacs.^22,28,29^

### Sequence Selection

**Figure 1:**
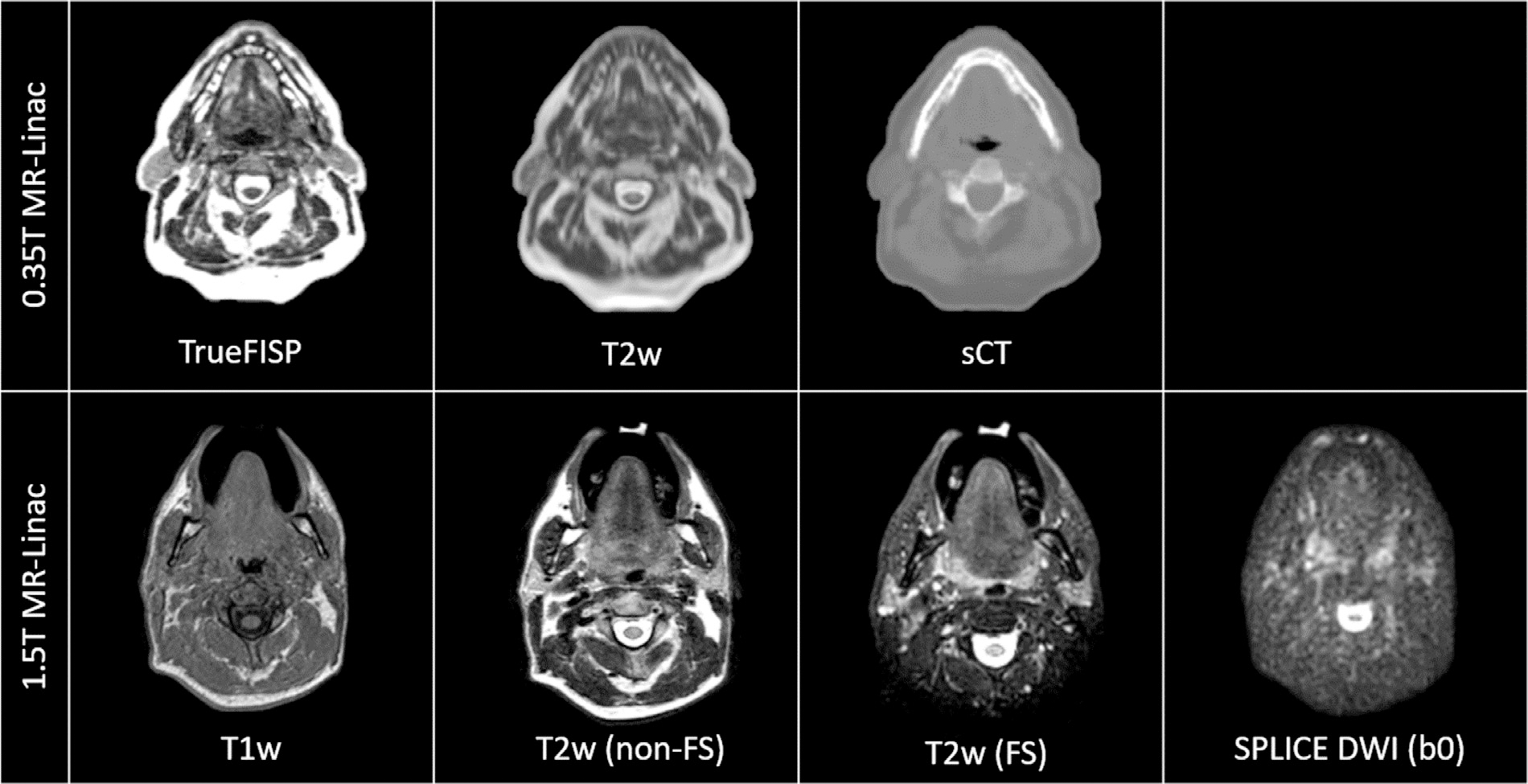
Selected sequences acquired on the 0.35T and 1.5T MR-linac systems. Abbreviations: TrueFISP, true fast imaging with steady-state free free precession; T1w, T1-weighted; T2w, T2-weighted; FS, fat-suppressed; sCT: synthetic CT; SPLICE, split acquisition of fast spin echo signal for diffusion imaging; DWI, diffusion-weighted imaging. (Color version of figure is available online.)

The primary sequences used for RT planning in MRgRT and for on-line treatment setup in MR-linac workflows are T1- and T2-weighted MRI sequences, which provide anatomical information about the size and shape of the tumors and organs at risk. In the first clinical workflow described for head and neck cancers on a commercial 1.5T MR-linac,^30^ 2 different 3-dimensional, turbo spin echo, non-fat suppressed, T2-weighted sequence were used: a 6-minute high-resolution/high-signal-to-noise ratio (SNR) scan for pre-treatment target delineation and when plans were adapted in the on-line “Adapt to Shape” workflow,^31^ and a 2-minute low-resolution/low-SNR scan for daily treatment setup and positioning in the on-line “Adapt to Position” workflow.^31^ Since the initial clinical implementation, the same group has transitioned to using a fat-suppressed version of the 6-minute T2-weighted scan for the same purposes due to the enhanced tumor contrast.^32^ In a 0.35T commercial MR-linac system, a 3-dimensional true fast imaging with steady-state free precession (TrueFISP) is used as the primary sequence for daily on-couch positioning,^33^ which is a fast imaging technique employing steady-state free precession imaging characterized by balanced gradients in all spatial directions with mixed T1- and T2-weighted signal contrast.^34^ The reported acquisition time of this sequence for head and neck applications is about 3 minutes,^35^ but recent efforts demonstrate that further reduction of acquisition time is still possible.^36^ In addition to anatomical sequences for daily setup and treatment plan optimization, both MR-linac systems also use 2-dimensional cine imaging for motion monitoring during beam delivery: a balanced fast field echo sequence on the 1.5T system^37^ and a TrueFISP sequence on the 0.35T system.^38^ Example images from the 0.35T and 1.5T systems are shown in Figure 1.

In a comprehensive review published by the DAHANCA group, the recommended MRI sequences for RT planning should be acquired in treatment position and include T1 with and without contrast enhancement and T2 with and without fat suppression.^7,8^ These sequences are commonly performed on MR simulator devices for RT planning, but they are not all currently available on existing MR-linac platforms. Furthermore, exogenous contrast is not routinely used with MR-linacs but is currently being investigated for safety and feasibility.^39–42^ Interestingly, intravenous contrast has not been shown to significantly affect the dose to target volumes or OARs in an MR-workflow for oropharyngeal cancers and could be used without dosimetric correction being necessary.^42^ Another possible class of sequences is the Dixon technique, which acquires an in-phase and an opposed-phase image that can be post-processed to separate signal from water and fat, resulting in more homogeneous fat suppression than other fat suppression techniques.^43^ Dixon sequences are already in use for segmentation and RT planning^44–46^ and can be used for additional evaluations, such as the longitudinal measurements of the water fraction in tissue^47^ or monitoring changes in the swallowing muscles.^48^

Furthermore, the use of functional MR imaging, particularly diffusion-weighted imaging (DWI) sequences, is emerging as a tool for RT treatment planning and response assessment in HNC. The image contrast in DWI is based on the restriction of the diffusion of water in tissues, which can be used to differentiate between malignant lesions and benign lesions/normal tissues due to the increased cellular density of tumors.^49–51^ When a tumor is treated with chemotherapy and/or RT, treatment-induced apoptosis can be reflected as an increase in the apparent diffusion coefficient (ADC) as measured by DWI.^52,53^ Several studies have shown that early changes in ADC from baseline can be used to predict treatment response, ^54–57^ leading to efforts to use DWI to monitor treatment response and adapt RT treatment plans based on early response.^58–61^ DWI can also be used in combination with anatomical sequences to assist target delineation and has been shown to lead to smaller GTVs and less inter-observer variability.^62,63^ However, compared to standard diagnostic MRI scanners, the lower field strengths and/or gradient strengths of the current commercially available MR-linac systems introduce additional challenges in acquiring DWI on MR-linacs.^64–66^ Still, recent data on DWI acquired with both systems has been promising, with studies showing adequate ADC accuracy and repeatability both in phantoms and *in vivo*.^64–73^

### MR-Based Planning and MR-Only Workflow

Although MRI simulation has been increasingly utilized in RT workflows, CT remains the primary imaging modality for simulation and treatment planning due to the quantitative electron density information needed for dose calculation. By incorporating methods to estimate electron densities on MRI such as bulk density assignment and synthetic CT (sCT), the requirement of a CT simulation could be eliminated, leading to an MR-only workflow. In addition to removing the cost and time burdens of the CT simulation, advantages of MR-only workflows include reducing patients’ exposure to ionizing radiation and eliminating any potential registration errors between the CT and MRIs.

Currently, the bulk density assignment is used on both commercially available MR-linac platforms.^74^ This method involves assigning a single representative electron density value to each voxel of a given structure; the selected densities can be taken from the mean value within the same structure on the patient’s planning CT if it is available, or they can be chosen from population reference values in an MR-only workflow. Although the present studies examining the accuracy of bulk density assignment in the head and neck region have used simplistic density assignments limited to soft tissue/water, bone, and air,^75–77^ the general consensus in the literature for head and neck cancers and other disease sites is that bulk density assignment results in clinically acceptable treatment plans with minimal dosimetric deviation from CT-based plans.^77–80^

The development of synthetic CT (sCT) from MRI is a further step towards establishing the primary role of MRI in treatment planning for the head and neck region. Several approaches have been proposed, but the seminal work of Han demonstrated the superiority of neural networks for this purpose.^81^ In a comparison of different deep-learning methods, generative adversarial networks (GANs) seem to be the most effective and are more commonly represented in the literature, although data for HNC are still scarce.^82^ Also, there are relatively few investigations about sCT generated from MRI in the head and neck region compared to sCT generated from CBCT or for PET attenuation corrections.^83^ Nonetheless, promising results for sCT have been reported,^84–87^ leading to the deployment of FDA approved commercial solutions. The quality of sCT for RT applications is commonly evaluated in retrospective analyses with respect to the planning CT,^82,88^ comparing either image-to-image similarity quantitative parameters such as differences in HU value or, preferably, dosimetric differences between the dose calculations on sCT and CT, which have been demonstrated to satisfy the 2% deviation recommendation for clinical implementation.^89^

Furthermore, the implementation of an MR-only workflow aims to eliminate the intrinsic uncertainty associated with the MR-CT registration.^90^ Performing both simulation and treatment at an MR-linac provides a single image modality for treatment planning and daily patient positioning, therefore eliminating the registration uncertainty with the CT, and allowing the sCT used for dose calculation to better reflect the patient’s anatomy at the time of setup.^88^ Finally, it should be noted that in cases where MR-only planning is used but treatment is delivered on a conventional linac (ie, non-MR-linac), further evaluation of the registration uncertainty between sCT and CBCT or kV imaging must be performed.^91^

### MR-Guided Adaptive RT (ART)

Head and neck cancers are susceptible to anatomical changes during the multiple weeks of treatment,^92,93^ and ART aims to compensate for such differences by modifying the treatment plan at least once throughout a course of RT.^94^ In addition to changes in the size and shape of the tumor throughout RT, ART can account for deformation of surrounding normal tissue caused by weight loss, tumor response, and radiation-induced damage to normal tissues. For example, high-frequency MR imaging during RT has shown that the parotid and submandibular glands can both migrate and shrink over the treatment course.^95^ In conventional non-adaptive treatments, healthy tissues can migrate into the high-dose regions, causing them to be irradiated to higher doses than indicated on the treatment plan because it was planned on the pre-treatment anatomy. Thus, by accounting for these anatomical changes, which are prevalent in the head and neck region, ART can potentially reduce radiation-induced side effects and improve long-term quality of life for HNC patients.

With its excellent soft tissue contrast, MRI can facilitate ART for HNC by enhancing visualization of both the tumor and normal tissues to enable mid-therapy adaptation. MR-guided ART (MRgART) can be accomplished either with conventional linacs aided by off-line MR simulations for re-planning or with hybrid MR-linacs that use on-board MRI and fast on-line re-planning. When conventional linacs are used, patients must undergo an additional MR simulation (and CT simulation if not using an MR-only workflow), and the new plan must be created off-line using the usual segmentation, planning, and plan review/quality assurance workflow. This process is time consuming for both patients and clinic staff and is often not feasible outside of specialized academic cancer centers. MR-linacs can streamline the MRgART process by acquiring the MRI for treatment planning and completing the planning and plan review all while the patient remains on the table in the treatment position. Although the total treatment duration is generally longer on an MR-linac (approximately 45–60 minutes) than on a conventional linac,^30^ treatment plans can be adapted as often as needed without separate simulation appointments and lengthy planning and quality assurance processes.

**Figure 2:**
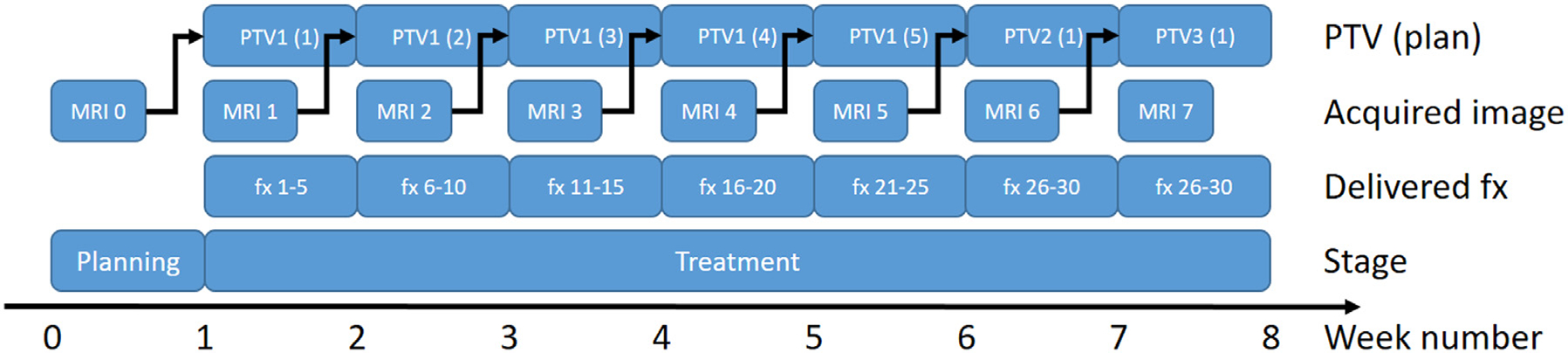
Overview of the workflow for weekly adaptation of head and neck treatment plans with regular acquisition of MRI at the MR-Linac. Figure adapted from van Timmeren et al.^100^. (Color version of figure is available online.)

While MRgART has been proposed as a means to mitigate acute and late effects,^96^ clinical evidence for the ability of ART in reducing side effects is still scarce, and the optimal timing and frequency of adaptive re-planning has not yet been determined. Clinical trials and retrospective studies aim to quantify the benefits and establish best practices. Several MRgART strategies are being investigated on both conventional linacs and MR-linacs, encompassing a range of fixed-interval adaptations as well as approaches with no pre-planned adaptation schedule where adaptation is triggered by some anatomical or dosimetric threshold.^30,97–101^ (NCT03972072). Figure 2 shows an example schedule of a weekly plan adaptation strategy implemented on a 0.35T MR-linac.^100^ While the technical feasibility of weekly adaptation has been demonstrated for head and neck cancers,^100^ high-frequency plan adaptations are limited by the time requirements for both MR-linac and conventional linac MRgART workflows. On MR-linacs, segmentation and contour editing on daily images is often a lengthy process due to both the large number of structures in the head and neck region and the poor performance of the current commercial deformable image registration-based contour propagation algorithms.^30,102,103^ With MRgART on conventional linacs, high-frequency adaptation poses challenges due to the process requiring a new simulation, adaptation of contours, replanning, plan review, and quality assurance in a very short time frame. Both workflows can be significantly expedited with automation and further refinement of the various steps of the re-planning processes, which are areas of active research but not yet fully realized in clinical practice. Ultimately, further evidence on the clinical benefit to patients will be required to evaluate the optimal frequency and time point(s) of adaptation and whether the additional time and costs associated with MRgART are justified.^104^

## On-Table Adaptation With MR-Linacs

The general on-line workflows for the two commercial MR-linac systems, utilizing 1.5T and 0.35T magnetic fields, have been discussed extensively in prior publications,^31,105–108^ so this section will focus primarily on considerations specific to HNC. A small number of studies have been published to date describing clinical workflows and initial outcomes for HNC on both MR-linac systems.^30,35,74,97,100^ In this section, we will summarize the reported workflows and offer insight into future directions to improve on-line MRgART for HNC.

**Figure 3:**
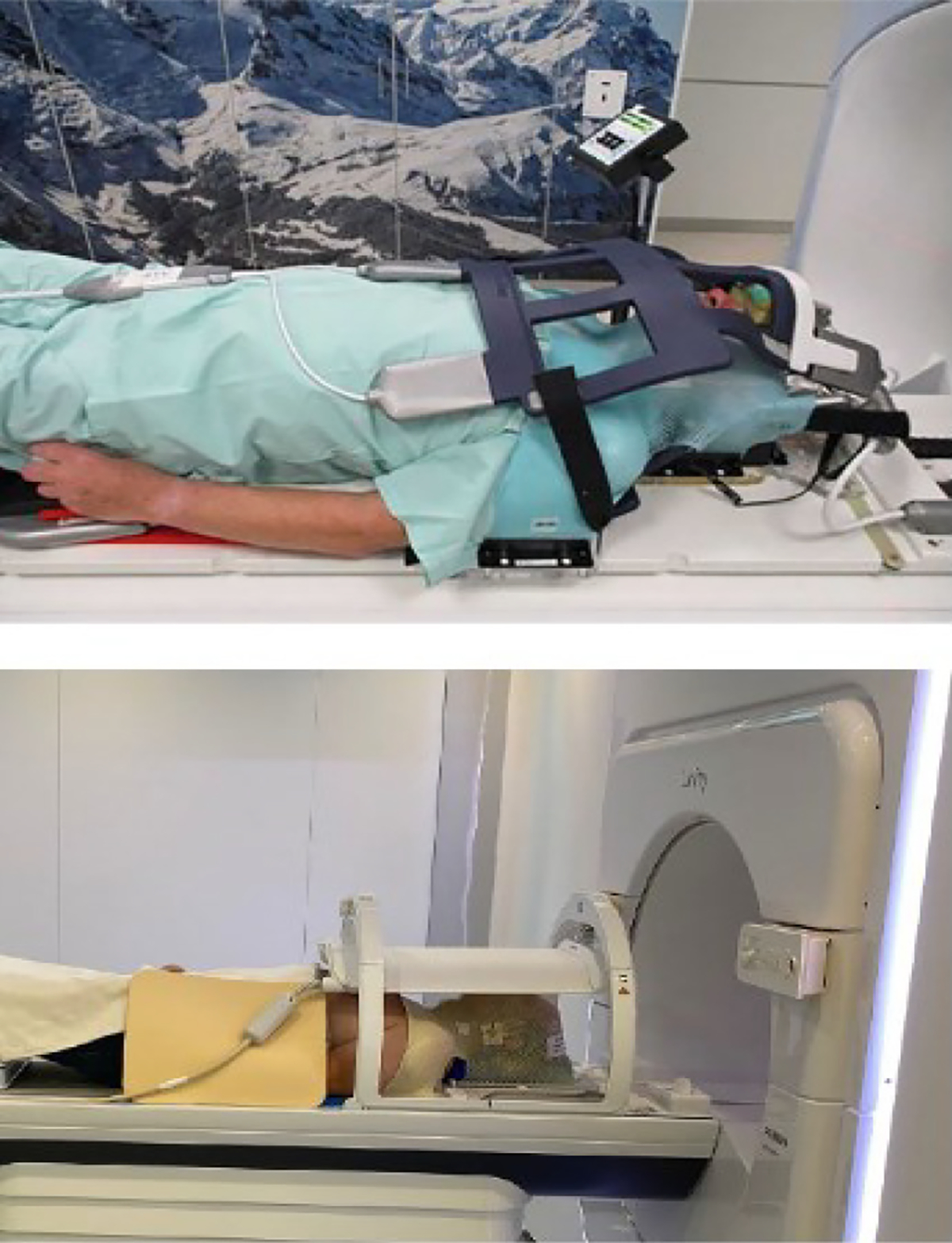
Patient immobilization and coil setup in the 0.35T (top) and 1.5T (bottom) MR-linac systems. Figure reproduced from Boeke et al.^111^. (Color version of figure is available online.)

As with conventional RT workflows, patients undergo pre-treatment simulation and IMRT treatment planning to create the reference plan. In all of the published HNC studies, patients received both a CT and an MR simulation.^30,35,74,97,100^ Patients are positioned in a custom head and neck immobilization mask. The treatment setup on both devices requires incorporation of the MRI receive coils into the setup (Fig. 3). On the 1.5T system, the posterior coil segment is incorporated into the table, and the rigid anterior coil is suspended above the patient from a ring.^109^ The 0.35T system uses two flex coils, one placed between the table and the patient’s head and one placed above the head.^110^ The maximum super-inferior field sizes are 22 cm and 24 cm for the 1.5T and 0.35T systems, respectively, which poses restrictions for the HNC patients who can be treated on these devices. Patients with multiple involved lymph node levels and/or with cancers of the nasopyarynx or nasosinus, which would require larger field sizes, may not be eligible. ^111^

Vendor-specific on-line workflows vary in specific details, but the general frameworks are the same. During each treatment, the patient is scanned with an anatomical MRI sequence in the treatment position: a combined T1/T2-weighted TrueFISP sequence for 0.35T system and a T2-weighted turbo spin echo sequence for the 1.5T system. This daily scan is then registered with a reference image from a previously created reference plan to determine the isocenter shift. The 0.35T system uses a physical couch shift,^108^ while the 1.5T system incorporates the isocenter shift into the new treatment plan as a virtual isocenter shift in a later step.^31^ Next, segmentation of daily images and adaptive treatment planning are performed; the options for re-planning will be discussed in more detail in the next paragraph. Finally, the treatment is reviewed and delivered if acceptable.

The segmentation and re-planning workflows differ between the two systems, and both systems offer multiple workflow options. In the 0.35T system, contours are propagated via rigid or deformable image registration from the reference image to the daily image, and the contours are manually edited if needed. Next, electron densities are assigned for dose calculation using bulk densities. The reference plan is then overlaid on the daily image, and the dose distribution is recalculated on the daily anatomy so that the clinician can decide whether to treat with the original plan or to adapt the plan.^108^ If the plan is adapted, it is reoptimized based on the current anatomy. In the 1.5T system, the clinician selects either the Adapt to Position (ATP) or the Adapt to Shape (ATS) workflow.^31^ In ATP, the virtual isocenter shift is applied to the reference plan to account for the change in target position. The daily setup image is not segmented and is used only to determine the virtual isocenter shift. The dose from the reference plan can be recalculated to account for the isocenter shift, or the reference plan can be reoptimized; in either case, the dose is calculated on the reference image rather than the current anatomy. In ATS, the contours are propagated from the reference image to the daily image using either rigid or deformable registration then edited if necessary. Bulk density assignment is used to transfer electron densities for dose calculation, then the treatment plan is reoptimized on the daily image.

The specific on-line adaptive workflows used for HNC patients have varied among published studies. In 2 reports by Chen et al. ^35,97^ with the 0.35T MR-linac, most patients were treated with the reference plan during each fraction without adaptive re-planning. However, according to the authors, these patients still benefited from treatment on the MR-linac because the improved soft tissue visualization enabled a reduction of PTV margins, which helped limit dose to organs at risk.^97^ In another study by van Timmeren et al.^100^ on the 0.35T system, plans were reoptimized off-line once per week during RT. For each re-plan, the tumor and organs at risk were segmented on a recent on-board MRI using deformable contour propagation and manual editing, then the reference plan was reoptimized according to the updated anatomy. That plan then served as a new reference plan for the following 5 fractions. In a similar approach implemented on the 1.5T system, McDonald et al. ^30^ described an off-line ATS/on-line ATP where an ATS plan was created off-line using a prior on-line image and used as a reference plan for ATP during subsequent treatments. This approach was chosen due to the poor quality of deformably propagated contours ^30,102,103^ and the extensive time required for manual correction of the many structures in the head and neck.^30^ Another approach proposed by Gupta et al.^74^ was the “ATS-Lite” method, which used the on-line ATS workflow during every treatment. However, instead of using deformable image registration to propagate all contours, it was only used for the external patient contour, and rigid registration was used for all others. This workflow eliminated the need for a physician to be present at each treatment for manual editing and review of contours but instead enabled off-line review once per week.

## Dose Accumulation

Currently, MR-linac platforms perform ART essentially on a fraction-by-fraction basis without accounting for the total delivered dose across all fractions. Not only are new treatment plans created throughout a single course of RT, but the patient anatomy changes as well. Dose accumulation is a relatively broad term but generally refers to approaches that integrate deformable image registration and deformable dose mapping between different time points to sum the individual plan doses and calculate delivered dose while accounting for anatomical changes and/or multiple treatment plans.^112,113^

Although dose accumulation is not yet widely used in MRgART and is not yet integrated into commercial MR-linac treatment planning systems, there are several reasons why dose accumulation can be valuable for clinical decision making and evaluation of patient outcomes.^114^ First, as MRgART enables the delivery of complex, individualized treatment regimens, dose accumulation can inform clinicians as to whether the intended goals of treatment are being met. It can also be used to quantitatively assess the safety and efficacy of different ART strategies, including varying the frequency and time point of plan adaptation and/or employing dose escalation or de-escalation approaches. Patient outcomes can be related to the total delivered dose rather than the planned dose to more rigorously assess the effects of these adaptive interventions. Furthermore, mid-RT adaptive plans can also potentially be optimized by accounting for the total dose delivered across all previous fractions. Finally, the existing normal tissue complication probability and tumor control probability models are based only on pre-treatment planned dose, but dose accumulation may introduce opportunities to refine these models and update treatment planning constraints for ART.^115,116^ However, it must be noted that extensive validation and estimation of uncertainty in the DIR and dose summation steps are critical for clinical implementation of any dose accumulation strategy.^112,117^

There are several considerations for dose accumulation unique to MRgART and MR-linac platforms, which have been discussed in detail by McDonald et al.^114^ One such consideration is that the quality of intra-modality deformable image registration is generally better than that of inter-modality deformable image registration,^30,102^ which supports the use of an MR-only workflow so that the daily images can be registered to the MR simulation image rather than CT. Also, in workflows where contours are not propagated to the daily image during the on-line workflow such as the 1.5T system ATP workflow, contours can be autosegmented and/or manually corrected on the daily image post-treatment and the delivered dose can be recalculated on the daily image prior to dose mapping and summation for a more accurate post-treatment evaluation. However, more research is needed to determine whether the small dose calculation accuracy improvement is worth the additional time burden. Next, the use of cine MRI sequences during beam delivery on MR-linacs enables intra-fraction dose accumulation to be post-treatment.^118^ Although immobilization masks limit intra-fraction anatomical motion in the head and neck region, several studies have demonstrated that respiratory and swallowing motion can cause periodic tumor motion, which can be especially severe for laryngeal cancers.^119,120^ In a study evaluating the dosimetric impact of swallowing motion, swallowing was infrequent enough for most patients to not impact delivered dose but could reduce dose to the planning target volume dose by up to 10% for patients with frequent, long swallows.^121^ Thus, intra-fraction dose accumulation strategies may be of interest for HNC.

## MRI Biomarkers and Radiomics

ART in general takes into account systematic and random variations through regular image feedback and leads to customization of the treatment plan, allowing for individualization of treatment.^94^ There exist multiple clinical goals of ART, which of course also apply to MRgRT and/or could potentially be improved by MRI, including (1) confirming accuracy of application and consistency of the original prescription^30^; (2) improving sparing of organs at risk^122^; and (3) biologically adaptive treatments, which could also facilitate dose (de-)escalation strategies, dose-painting, spatio-temporal fractionation, and individually tailored treatments.^101,123,124^ The first 2 aspects were sufficiently covered in the sections above. The present section focuses on the use of quantitative features of MRI as biomarkers to guide planning and adaptation.^59^ MRI biomarkers can be derived either from quantitative MRI techniques such as DWI or dynamic contrast-enhanced (DCE) MRI or from radiomics analysis, a machine learning technique used to extract textural information from pixels. At present, relatively few studies have evaluated the use of MRI biomarkers in HNC, and most of them have used diagnostic platforms and small patient cohorts. Moreover, no established reliable MRI biomarker exists that fulfills all of the international recommendations for clinical adoption.^125,126^ This can be attributed not only to the novelty of the topic and the relatively recent feasibility of repeat and/or on-line imaging, but also to the lack of standardization. Table summarizes the existing literature on the use of quantitative MRI biomarkers for prediction in HNC.

### MRI Biomarkers for Tumor Response

As presented in Table, diffusion-weighted imaging is the most commonly used modality for response prediction in HNC. Several studies have demonstrated the value of this modality in predicting tumor response in primary tumors and/or lymph nodes, correlating quantitative MRI sequences acquired before treatment with outcomes and often comparing them with imaging during and after the end of treatment. The apparent diffusion coefficient (ADC) is the most thoroughly investigated parameter in these series with both the pre-treatment values and longitudinal changes seeming to be associated with locoregional control and survival.^149^ The ADC is a parameter that quantifies the Brownian movement of water molecules in tissue. In a systematic review by Martens et al.,^149^ early increases in ADC during chemoradiotherapy (ie, changes from baseline to the second or third week of treatment) were significantly predictive for locoregional control. Other functional MRI modalities like DCE imaging, amide proton transfer-weighted (APTw) imaging, and spectroscopy have been also studied at similar time points, showing promising results but only in few and small cohorts.^136,137,141,142^ These methods quantify physiological features such as kinetics and perfusion (DCE), or the content of mobile proteins and peptides (APTw).

The advent of machine learning allowed the development of models to analyze tumor heterogeneity in 3-dimensional models extracting features from diagnostic imaging, so called “radiomics.” As this AI-based technology is still in its infancy, there are major challenges to overcome, like image artifacts, interpretability of the models, variability in tumor segmentation, standardization of imaging techniques, and cross-center validation.^150^ In one of the newest and largest studies published so far, Mes et al.^148^ extracted over 500 radiomic features in 4 different cohorts of patients with oral or HPV-negative oropharyngeal cancers and successfully developed prediction of recurrence and survival models that outperformed classical clinical feature-based models. These data originated from different MRI vendors and imaging protocols, which was encouraging, but reproducibility has yet to be proven. Although some larger, multi-center cohorts have been published for CT radiomics, the few series investigating MR radiomics have been strictly retrospective and mostly lack external validation. However, very recently, a multi-center consortium validated a radiomic signature based on T1 and T2 sequences that increased the prognostic ability of clinical parameters, both for HPV-positive and HOV-negative HN.^151^

Importantly, nearly all of the aforementioned MRI biomarker studies published so far have been performed on classical diagnostic MRI platforms with 1.5 or 3 T magnetic fields, with one exception, in which the 0.35T MR-linac system was utilized.^68^ Additionally, two recent studies quantified the test-retest repeatability of ADC in HNC on the 1.5 T MR-linac and found that the system demonstrated acceptable repeatability performance in this disease site.^71,72^ Integrating MR-linacs in future biomarker studies will facilitate development of large databases including longitudinal imaging at all possible time points during treatment, allowing for even daily comparability and monitoring, perfectly integrated in the daily workflows.

### MRI Biomarkers of Normal Tissue Injury

Data regarding MRI biomarkers for prediction and evaluation of radiation-induced toxicity are even more scarce. A few studies so far have implemented sequential MRI for describing changes in the salivary glands.^152–155^ These studies used mostly diagnostic MRI platforms at 2 different time-points, for example, DWI- and DCE-sequences for assessing changes in the parotids. Van Timmeren. et al.^100^ were the first to prospectively utilize weekly MRI to demonstrate changes in all 4 major salivary glands (parotids and submandibular glands) during the whole course of the treatment on an MR-linac. The improved dosimetry occurring from such longitudinal, adaptive approaches—especially if doses are accumulated—could improve xerostomia prediction and development of more accurate normal tissue complication probability models.^115,116^ Another added value of this approach would be the discovery of novel biomarkers to predict toxicity.

Some of the functional imaging methods used for prediction of tumor control have already been evaluated also for quantifying normal tissue injury: DCE-changes, for example, are a direct correlate for injuries in normal vascular structures^156^ and are associated with increased rates of osteoradionecrosis of the jaw. ^157,158^ Relaxometry, a method of characterizing tissues by quantifying T1 and T2 relaxation coefficients, has been used as a measure of inflammatory reaction and edema of the pharyngeal constrictor muscles and linked to dysphagia.^159^ These changes in the pharyngeal musculature have been shown to be dose-dependent.^160^ Similar studies have investigated relaxometry-sequences, so called “T1rho,” for evaluating changes in the parotid glands and prediction of xerostomia.^161,162^ These MRI biomarkers allow for an early, noninvasive prediction of xerostomia with the possibility of easy integration in adaptive MRgRT-workflows.

Xerostomia is the most commonly studied side effect for HNC with MRI radiomics. Van Dijk et al.^163^ found that the pre-treatment fat-to-functional tissue ratio of the parotid glands of 68 patients was associated with xerostomia at 12 months after treatment. Sheikh et al.^164^ came to similar results when evaluating baseline extracted features in the parotids of 266 patients and correlating those with xerostomia 3 months after irradiation. Such approaches can help to identify patients with increased risk of xerostomia and individualize treatments to improve quality of life of long-time survivors. There exist almost no more MRI radiomics data for evaluating radiation induced injury of other organs or correlation with other sequela. One small retrospective study showed a clear dose dependence of contrast-enhanced T1 textures in the masseter and pterygoid muscles and clinical manifestation of trismus within the first year following IMRT.^165^ Currently, unlike for other MRI biomarkers, there is a lack of studies with machine learning models based on repeat imaging and longitudinal texture extraction during the whole treatment course, something which could also be improved with daily on-line imaging on an MR-linac. In general, toxicity predicting radiomics models are subject to the same underlying limitations as the ones for predicting tumor control, and introduction to the clinic will only be possible after large, prospective cross-center validation studies.^150^

### Standardized Reporting of MR-Guided Adaptive Interventions for HNC

At present, the admittedly limited data from prospective trials of ART (either CT, PET, or MR-guided) has occurred in the context of relatively simple, often single-timepoint adaptation schedules. However, the relative ubiquity of high-frequency multi-parametric imaging now available on hybrid MR-linac devices allows a markedly more varied degree of applied adaptation, a veritable flood of information. In a previous report, Heukelom and Fuller defined a usable terminology for describing clinical dosimetric intent and a workable descriptive typology for adaptive implementation notation.^101^ Increasingly complex trial processes (eg, use of functional and anatomic imaging, variable dose-of-the day methods, and innovative stereotactic approaches) have expanded the complexity of MRgART methods. However, there is not yet a standard for reporting specific elements of adaptive treatment regimens to enable direct comparison between studies and evaluation of various ART schemas.

For this reason, the American Society of Radiation Oncology has convened a consensus panel group, which in 2023 began the task of defining standardized guidance for MR-guided adaptive therapy; simultaneously, American Association of Physicists in Medicine efforts such as Task Group No. 384 (“Clinical Implementation of Automated Segmentation for Adaptive Radiation Therapy (ART)”), and Task Group No. 352 (“MR-guided Radiotherapy Systems: Considerations for Clinical Implementation and Quality Assurance”) have efforts under way that will likely serve as definitive guidance for reporting of MRgART studies. However, in the interim, in an effort to serve the large community, we have developed a draft template for recommended components for manuscript reporting of MR-guided adaptive therapy (Appendix). This questionnaire addresses topics such as the clinical dosimetric intent of ART, the frequency and/or triggers of adaptation, images used for pretherapy planning and adaptation, segmentation of target volumes and organs at risk for pretherapy planning and adaptation, dosimetric constraints, on-line vs. off-line ART, and dose accumulation. While necessarily preliminary given ASTRO and AAPM efforts, this template serves as corollary reporting questionnaire which can be submitted with manuscripts in order to ensure clarity and efficacy in description of MR-guided clinical trials and provide sufficient descriptive rigor to allow extramural replication of clinical reports.

## Summary and Conclusions

In this review, we summarized the existing clinical experience and research developments with MRgRT for HNC, including both off-line adaptive approaches and on-line MRgART with MR-linac devices. The integration of MRI into the treatment planning and/or ART processes for HNC has expanded technical capability but has also introduced additional challenges and considerations unique to MRI. The ability to acquire a variety of both anatomical and functional MRI sequences at a high frequency throughout RT enables evaluation of both tumor response and normal tissue function and allows us to adapt treatments based on anatomical and physiological changes. While MRgRT enables a range of potential adaptive treatment techniques, no standardized reporting mechanism currently exists to describe adaptive treatments. In this article, we provide a template for reporting MRgART treatments to promote wider clinical adoption and enable the production of strong clinical evidence for MRgART.

## Figures and Tables

**Table:** Overview of Selected Published Series Implementing Predictive MRI Biomarkers in HNC

Lead Author	Patients (n)	Platform	Technique/ Sequence	Timepoints

Hoang^127^	16	diag, 1.5T	DWI	3: 2x pre-treatment, then after 2nd w of CRT
King^128^	30	diag, 1.5T	DWI	2: pre-treatment and 2nd w
Schouten ^129^	8	diag, 1.5T	DWI (EPI+HASTE)	3: pre-treatment, 14th d and after 3 mo
Galban ^130^	15	diag, 3T	DWI	2: pre-treatment and 3rd w
Wong ^131^	35	diag, 1.5T	DWI + DCE	3: pre-treatment and 1st and 2nd w
Kim ^132^	33	diag, 1.5–3T	DWI	3: pre-treatment, 1st w and 1 w after CRT
Hatakenaka^133^	17+40	diag, 1.5T	DWI	3: pre-treatment, 7th d and in FU
Matoba^56^	40	diag, 1.5T	DWI	2: pre-treatment and 3rd w
Yang^68^	6	MRL, 0.35T	DWI	4–7: every 3–5 d during CRT
Lambrecht^58^	20	diag, 1.5T	DWI	2: pre-treatment and 2nd w
Vandecaveye ^54^	30	diag, 1.5T	DWI	3: pre-treatment, 2nd w and 3rd w
von der Grun ^134^	17	diag, 1.5T	DWI	3: pre-treatment, 15th d and FU 6–8w
Noij^135^	78 (retr)	diag, 1.5T	DWI	1: pre-treatment
Baer ^136^	10	diag, 3T	DCE	2: pre-treatment and 3rd w
Wang ^137^	14	diag, 3T	DCE	2: pre-treatment and 2nd w
Marzi ^138^	34	diag, 1.5T	IVIM-DWI	3: pre-treatment, mid-treatment, after
Paudyal ^139^	34	diag, 3T	IVIM-DWI	4: pre-treatment, 1st, 2nd, 3rd w
Hauser ^140^	22	diag, 3T	IVIM-DWI	2: pre-treatment and FU
King ^141^	60	diag, 1.5T	cholin-spectroscopy	2: pre-treatment and 2nd w
Qamar ^142^	16	diag, 3T	APTw	2: pre-treatment and 2nd w
Scalco ^143^	30	diag, 1.5T	Radiomics (T2)	2: pre-treatment and mid-treatment
Yuan ^144^	85+85 (retr)	diag, 1.5T	Radiomics (T2)	1: pre-treatment
Siow ^145^	198 (retr)	diag, 3T	Radiomics (T1-CE)	1: pre-treatment
Bos ^146^	157 (retr)	diag, 1.5T	Radiomics (T1-CE)	1: pre-treatment
Zhong ^147^	1872 (retr)	diag, 1.5–3T	Radiomics (T1, T2, T1-CE)	1: pre-treatment
Mes^148^	102+76+89+56	diag, 1.5–3T	Radiomics (T1)	1: pre-treatment

Abbreviations: Retr, retrospective; diag., diagnostic platform; MRL, MR-Linac; DWI, diffusion weighted imaging; DCE, dynamic contrast-enhanced; APTw, amide proton transfer-weighted; CE, contrast-enhanced; w, week(s); IVIM, intravoxel incoherent motion; d, day(s); mo, months; CRT, chemoradiotherapy; FU, follow up.

## Appendix

Standard Reporting Considerations for Adaptive MRI-guided SBRT for Head and Neck Cancers:

1. What is the clinical dosimetric intent of the proposed adaptive regimen?
  Choose all that apply:
  - Preservation of initial planned dose at simulation
  - Reduction in GTV/CTV volume with shrinking tumor
  - Pre-specified dose escalation to a image-defined sub-volume
  - Isotoxic dose escalation to pre-defined normal tissue threshold
2. What is the frequency of planned adaptation?
  Choose all that apply:
  - Fixed interval (every ____ fractions)
  - Triggered interval (using pre-specified dosimetric or volumetric thresholds/indicators)
    - Specify triggering events for re-planning
  - Ad hoc (unscheduled, upon physician/physics request)
3. What immobilization strategy/devices are implemented at simulation? Are they standardized for all patients in the regimen/trial?
4. Describe any reference anatomic (T1W, T2W) and/or functional imaging used for adaptation.
  - Specify whether images are used for alignment/positional verification, dose calculation/pseudo-CT generation, and/or for target delineation.
  - Specify sequences using both vendor-supplied sequence version, as well as generic descriptors (eg, fat suppression, fluid suppression)
    - Specify standard acquisition parameters
      1. Acquisition type (2D/3D)
      2. Acquisition orientation (axial/coronal/sagittal/oblique)
      3. TE
      4. TR
      5. Excitation flip angle
      6. RF pulse train flip angle pattern
      7. Oversample factor
      8. FOV (AP × RL × FH)
      9. Acquisition voxel size
      10. Reconstruction voxel size
      11. Number of signal averages
      12. Number of slices
      13. Reconstruction method (eg, SENSE, GRAPPA)
5. Is additional imaging (eg, MRI, PET, or CT) used for pretherapy treatment planning? If so, are all images archived and registered with the simulation DICOM dataset? Please specify whether used for target delineation, dose calculation, and/or therapy response. If post-processing is performed (eg, quantitative maps such as ADC, K_trans_, SUV), please specify software/method used.
  - Simulation images (in therapy position)
    - MR
      1. Specify sequences
      2. Specify contrast agent
    - CT
      1. Specify contrast agent
    - PET
      1. Specify tracer
6. Are ROIs (TVs/OARs) segmented manually or using automated/semi-automated processes? If so, is patient-specific quality assurance of initial segmentation volumes performed, and if so, by whom?
7. What quality assurance procedures, guidelines, and nomenclature are used in the treatment planning system for description of TV/OAR ROIs?
8. What are the pretherapy dose-constraints implemented for TVs/OARs? Are any constraints based on a biological model? If so, please provide specific citations.
9. Is the on-treatment re-imaging performed on-line or offline? What is the frequency of on-treatment re-imaging? Are all on-treatment images archived?
10. Are additional off-line image data implemented (eg, contrast MRI, PET, diagnostic CT), and if so, how are they utilized (eg, synthetic CT or registration-based dose calculator, target delineation, etc.)? Are all utilized off-line images archived with co-registration to the closest interval on-line volumetric images?
11. Is the re-planning strategy on-line or off-line? What is the frequency/interval of adaptive re-planning? What software/version/algorithm is utilized for re-planning/adaptation? Please list software/version used for all cases.
12. What are the re-planning criteria/action level specified? Are non-dosimetric surrogate criteria (eg, weight loss) used as a trigger for re-planning?
13. Are ROIs for adaptation (re)segmented manually, automatically, or semi-automated, or rigid or DIR-propagated? Is faculty/staff approval required for relevant ROIs, and if so, are these annotated and timestamped?
14. Is dose accumulation performed, and if so, how (eg, iterative dose-accumulation or superimposition of delivered dose)? What software/version/algorithm is used for initial and re-planning dose calculation? Are final accumulated dose and back-projected dose archived/summarized? Is interval dose assessment incorporated into re-planning decisions, and if so, how?

### Special Technical Considerations for Adaptive MRI-guided SBRT for Head and Neck Cancers:

1. How are motion management and intrafraction motion monitoring addressed during adaptive MRI-guided SBRT for head and neck cancers?
2. What immobilization strategies and devices are specifically used for head and neck SBRT to ensure reproducibility?
3. How are the dose constraints for OARs and PTVs modified to reflect the SBRT treatment approach?
4. Are there any specific considerations in the MRI-guided SBRT workflow, such as the integration of functional imaging or the use of gating techniques?
