# Corrigendum to “The Use of MR-Guided Radiation Therapy for Head and Neck Cancer and Recommended Reporting Guidance” Seminars in Radiation Oncology Volume 34 (2024) 69–83

**DOI:** 10.1016/j.semradonc.2024.04.001

**Published:** 2024-07

**Authors:** Brigid A. McDonald, Riccardo Dal Bello, Clifton D. Fuller, Panagiotis Balermpas

**Affiliations:** * Department of Radiation Oncology, The University of Texas MD Anderson Cancer Center, Houston, TX; † Department of Radiation Oncology, University Hospital Zurich and University of Zurich, Zurich, Switzerland

The authors regret that the grant support and conflicts of interest for some authors were mistakenly omitted in the initial publication. The following acknowledgements and disclosures will be added:

Grant support:

Dr. McDonald receives funding and salary support from an Image Guided Cancer Therapy (IGCT) T32 Training Program Fellowship (T32CA261856) and an ASTRO-AAPM Physics Resident/Post-Doctoral Fellow Seed Grant. Dr. Fuller received/receives related funding and salary support from: National Institutes of Health (NIH) National Cancer Institute (NCI)/National Science Foundation (NSF) Smart Connected health Program (R01CA257814); NIH National Institute of Dental and Craniofacial Research (NIDCR) Academic Industrial Partnership Grant (R01DE028290); and NIDCR Establishing Outcome Measures for Clinical Studies of Oral and Craniofacial Diseases and Conditions award (R01DE025248). Dr. Fuller receives relevant grant and infrastructure support from MD Anderson Cancer Center via the Charles and Daneen Stiefel Center for Head and Neck Cancer Oropharyngeal Cancer Research Program; the Program in Image-guided Cancer Therapy; and the NIH/NCI Cancer Center Support Grant (CCSG) Image-Driven Biology-Informed Therapy (IDBT) Program (P30CA016672).

Conflicts of interest:

Dr. McDonald has received travel funding from Elekta AB. Dr. Fuller has received NIH sub-award support from Oncospace, Inc. (R43CA254559, PI Lakshminarayanan) under a Small Business Innovation Research Grant Applications grant. Dr. Fuller has received direct industry grant/in-kind support, honoraria, and travel funding from Elekta AB. Dr. Fuller has served in a consulting capacity for Varian/Siemens Healthineers, Philips Medical Systems, and Oncospace, Inc.

The authors would like to apologize for any inconvenience caused.

